# Molecular Classification of the PORTEC-3 Trial for High-Risk Endometrial Cancer: Impact on Prognosis and Benefit From Adjuvant Therapy

**DOI:** 10.1200/JCO.20.00549

**Published:** 2020-08-04

**Authors:** Alicia León-Castillo, Stephanie M. de Boer, Melanie E. Powell, Linda R. Mileshkin, Helen J. Mackay, Alexandra Leary, Hans W. Nijman, Naveena Singh, Pamela M. Pollock, Paul Bessette, Anthony Fyles, Christine Haie-Meder, Vincent T. H. B. M. Smit, Richard J. Edmondson, Hein Putter, Henry C. Kitchener, Emma J. Crosbie, Marco de Bruyn, Remi A. Nout, Nanda Horeweg, Carien L. Creutzberg, Tjalling Bosse, on behalf of the TransPORTEC consortium

**Affiliations:** ^1^ Department of Pathology, Leiden University Medical Center, Leiden, the Netherlands; ^2^ Department of Radiation Oncology, Leiden University Medical Center, Leiden, the Netherlands; ^3^ Department of Clinical Oncology, Barts Health National Health Service Trust, London, United Kingdom; ^4^ Department of Medical Oncology, Peter MacCallum Cancer Centre, Melbourne, Victoria, Australia; ^5^ Division of Medical Oncology and Hematology, Sunnybrook Odette Cancer Centre, Toronto, Ontario, Canada; ^6^ Department of Medical Oncology, Gustave Roussy, Villejuif, France; ^7^ Department of Gynecology, University Medical Center Groningen, University of Groningen, Groningen, the Netherlands; ^8^ Department of Pathology, Barts Health National Health Service Trust, London, United Kingdom; ^9^ Institute of Health and Biomedical Innovation, Queensland University of Technology, Brisbane, Queensland, Australia; ^10^ Canadian Cancer Trials Group, Department of Obstetrics and Gynecology, University of Sherbrooke, Sherbrooke, Quebec, Canada; ^11^ Canadian Cancer Trials Group, Radiation Medicine Program, Princess Margaret Cancer Centre, Toronto, Ontario, Canada; ^12^ Department of Radiotherapy, Gustave Roussy, Villejuif, France; ^13^ Division of Cancer Sciences, University of Manchester, St Mary's Hospital, Manchester, United Kingdom; ^14^ Department of Biostatistics, Leiden University Medical Center, Leiden, the Netherlands

## Abstract

**PURPOSE:**

The randomized Adjuvant Chemoradiotherapy Versus Radiotherapy Alone in Women With High-Risk Endometrial Cancer (PORTEC-3) trial investigated the benefit of combined adjuvant chemotherapy and radiotherapy (CTRT) versus radiotherapy alone (RT) for women with high-risk endometrial cancer (EC). Because The Cancer Genome Atlas defined an EC molecular classification with strong prognostic value, we investigated prognosis and impact of chemotherapy for each molecular subgroup using tissue samples from PORTEC-3 trial participants.

**METHODS:**

Paraffin-embedded tissues of 423 consenting patients were collected. Immunohistochemistry for p53 and mismatch repair (MMR) proteins, and DNA sequencing for *POLE* exonuclease domain were done to classify tumors as p53 abnormal (p53abn), *POLE-*ultramutated (*POLE*mut), MMR-deficient (MMRd), or no specific molecular profile (NSMP). The primary end point was recurrence-free survival (RFS). Kaplan-Meier method, log-rank test, and Cox model were used for analysis.

**RESULTS:**

Molecular analysis was successful in 410 high-risk EC (97%), identifying the 4 subgroups: p53abn EC (n = 93; 23%), *POLE*mut (n = 51; 12%), MMRd (n = 137; 33%), and NSMP (n = 129; 32%). Five-year RFS was 48% for patients with p53abn EC, 98% for *POLE*mut EC, 72% for MMRd EC, and 74% for NSMP EC (*P* < .001). The 5-year RFS with CTRT versus RT for p53abn EC was 59% versus 36% (*P* = .019); 100% versus 97% for patients with *POLE*mut EC (*P* = .637); 68% versus 76% (*P* = .428) for MMRd EC; and 80% versus 68% (*P* = .243) for NSMP EC.

**CONCLUSION:**

Molecular classification has strong prognostic value in high-risk EC, with significantly improved RFS with adjuvant CTRT for p53abn tumors, regardless of histologic type. Patients with *POLE*mut EC had an excellent RFS in both trial arms. EC molecular classification should be incorporated in the risk stratification of these patients as well as in future trials to target specific subgroups of patients.

## INTRODUCTION

The endometrial cancer (EC) molecular classification introduced by The Cancer Genome Atlas^1^ has initiated a transition toward molecular-based classification with clear prognostic value and thus a potential impact on the clinical care of patients with EC. The significant prognostic differences among the 4 molecular subgroups have been replicated using surrogate markers in formalin-fixed, paraffin-embedded (FFPE) tissues, identifying analogous subgroups: p53-abnormal (p53abn), *POLE-*ultramutated (*POLE*mut), mismatch repair–deficient (MMRd), and no specific molecular profile (NSMP) EC. The integration of the molecular classification with clinicopathological features has resulted in improved prognostic accuracy in intermediate-risk EC as well as unselected cohorts,^2-5^ highlighting the potential of the molecular classification to refine and further individualize patients’ risk stratification.

**Context**

**Key Objective**
To determine, using tissue samples from the PORTEC-3 clinical trial, the prognostic value of the endometrial cancer (EC) molecular classification in high-risk EC and the possible benefit from chemotherapy within each molecular subgroup.
**Knowledge Generated**
The molecular classification has a strong prognostic value in high-risk EC. Additionally, the molecular classification may guide adjuvant treatment decisions for patients with high-risk EC and supports adjuvant chemotherapy for p53abn ECs.
**Relevance**
This study shows that incorporation of the molecular classification into risk stratification systems is essential and future clinical trials should address specific molecular subgroups of EC. Adjuvant treatment decisions based on the molecular classification are supported; specifically, patients with p53abn EC should be considered for adjuvant chemoradiotherapy, whereas for those with *POLE*mut cancers, de-escalation of adjuvant treatment should be considered.

Although patients with EC generally have a good prognosis, 15%-20% have high-risk disease with increased incidence of distant metastases and cancer-related death. Characteristics defining high-risk EC are high-grade disease, advanced stage, and/or nonendometrioid histology. Adjuvant pelvic external beam radiotherapy (EBRT) is a standard of care for patients with high-risk EC.^6^ The randomized Adjuvant Chemoradiotherapy Versus Radiotherapy Alone in Women With High-Risk Endometrial Cancer (PORTEC-3) trial investigated the benefit of combined adjuvant chemotherapy and EBRT (CTRT) versus EBRT alone (RT) in patients with high-risk EC.^7^ PORTEC-3 showed a significant benefit in both overall survival (OS) and failure-free survival (FFS) with CTRT, although the absolute benefit was limited (5% for 5-year OS and 7% for 5-year FFS), and significantly more adverse events occurred with CTRT. The greatest benefit was observed in serous cancers and stage III disease.^8^ However, because there is substantial interobserver variability in assessment of pathologic factors that define high-risk, especially in high-grade EC,^9,10^ it remains a challenge to identify patients who will benefit from chemotherapy. In this context, the molecular classification might help determine appropriate adjuvant treatment.

Using tissue samples donated by PORTEC-3 clinical trial participants, we investigated the prognostic relevance of the molecular classification and the relationship between the molecular subgroups and benefit from adjuvant CTRT in patients with high-risk EC.

## METHODS

### Patient Selection and Study Design

FFPE tissue was collected from 423 consenting patients from 5 of the 6 clinical trial groups participating in the PORTEC-3 clinical trial.^7^ The design and results of the PORTEC-3 trial have been reported previously^7^ and are further described in the Data Supplement. Briefly, this international phase III trial enrolled patients with high-risk EC (endometrioid EC [EEC] grade 3 stage IA with documented lymphovascular space invasion [LVSI]; EEC grade 3 stage IB; EEC stage II-III; and nonendometrioid invasive EC stages I, II, or III). Upfront central pathology review was done by reference gynecopathologists to confirm eligibility.^11^ Abdominal or laparoscopic hysterectomy with bilateral salpingo-oophorectomy was performed on all patients. Patients were randomly allocated 1:1 to EBRT alone or CTRT (2 cycles of cisplatin followed by 4 cycles of carboplatin and paclitaxel). The study was approved by the Dutch Cancer Society and ethics committees of participating groups.

### Procedures

Immunohistochemical staining for p53 and MMR proteins (MLH1, PMS2, MSH2, and MSH6) was performed on all cases (see Data Supplement for procedures and scoring). If p53 immunohistochemistry was not evaluable, *TP53* mutational status was used (n = 9; 2.1% of the total 423 EC cases). If MMR immunohistochemistry was not evaluable, microsatellite instability (MSI) was assessed (n = 8; 1.9%) using the MSI analysis system, version 1.2 (Promega, Madison, WI).

DNA isolation was performed as described previously.^12^ *POLE* mutational status was assessed by next-generation sequencing using the AmpliSeq Cancer Hotspot Panel, version 5 (Thermo Fisher Scientific, Waltham, MA), a panel including *POLE* exonuclease domain and *TP53*, as described in the Data Supplement. If sequencing with the panel failed, KASPar competitive allele-specific polymerase chain reaction (LGC Genomics, Berlin, Germany) assays were used to screen for *POLE* hotspot variants at codons 286, 297, 411, 456, and 459 (primer sequences are available upon request). *POLE* exonuclease domain mutations (EDMs) were considered pathogenic (in this context, mutations causative of ultramutation) following previously defined criteria.^13^ Sequencing and immunohistochemistry results were evaluated blinded for patient outcome.

If ≥ 1 of the molecular features (p53, MMR, and/or *POLE* status) could not be determined (eg, not enough tumoral material or testing failed), and thus the molecular subgroup could not be determined, the case was classified as EC, not otherwise specified^14^ and excluded from the study (n = 13). Tumors with > 1 classifying feature (multiple-classifier EC) were allocated in 1 of the 4 molecular subgroups, as described previously.^13,15^ Briefly, ECs with a pathogenic *POLE* EDM with p53 abnormal expression and/or MMR protein loss were classified as *POLE*mut EC, whereas ECs with loss of any MMR protein or MSI-high with a p53 mutant staining pattern were classified as MMRd EC (Data Supplement).^13,15^

### Statistical Analysis

The primary end point was RFS, defined as time from randomization to date of first relapse or death, whichever occurred first. The secondary end point was OS, defined as the time from randomization to date of death of any cause.

Statistical analysis was performed using SPSS statistics, version 25 (IBM, Armonk, NY) and R, version 3.6.1). Clinicopathological characteristics of patients included in this study and the complete PORTEC-3 cohort, as well as between molecular subgroups, were compared with a χ^2^ test for categorical variables or 1-way ANOVA for continuous variables. All analyses were based on intention to treat. Analysis was also done by treatment received, without relevant differences (data not shown).

Differences in RFS and OS between molecular subgroups were tested with log-rank test and Cox regression analysis, which included molecular subgroups, age (as a continuous variable), histology (EEC grade 1-2 *v* EEC grade 3 or mixed *v* nonendometrioid serous carcinoma, clear-cell carcinoma, or other), stage (stage I-II *v* III), and adjuvant treatment received (CTRT *v* RT). To identify differences in treatment effect between molecular subgroups, Cox regression analysis was performed with treatment received, molecular subgroup, and their interaction. Similarly, Cox regression with treatment received, stage, and their interaction was performed within each molecular subgroup to analyze treatment effect per stage. Median follow-up time was calculated using the reverse Kaplan-Meier method. Reported *P* values were based on 2-sided tests, with *P* < .05 considered statistically significant.

## RESULTS

**FIG 1.**
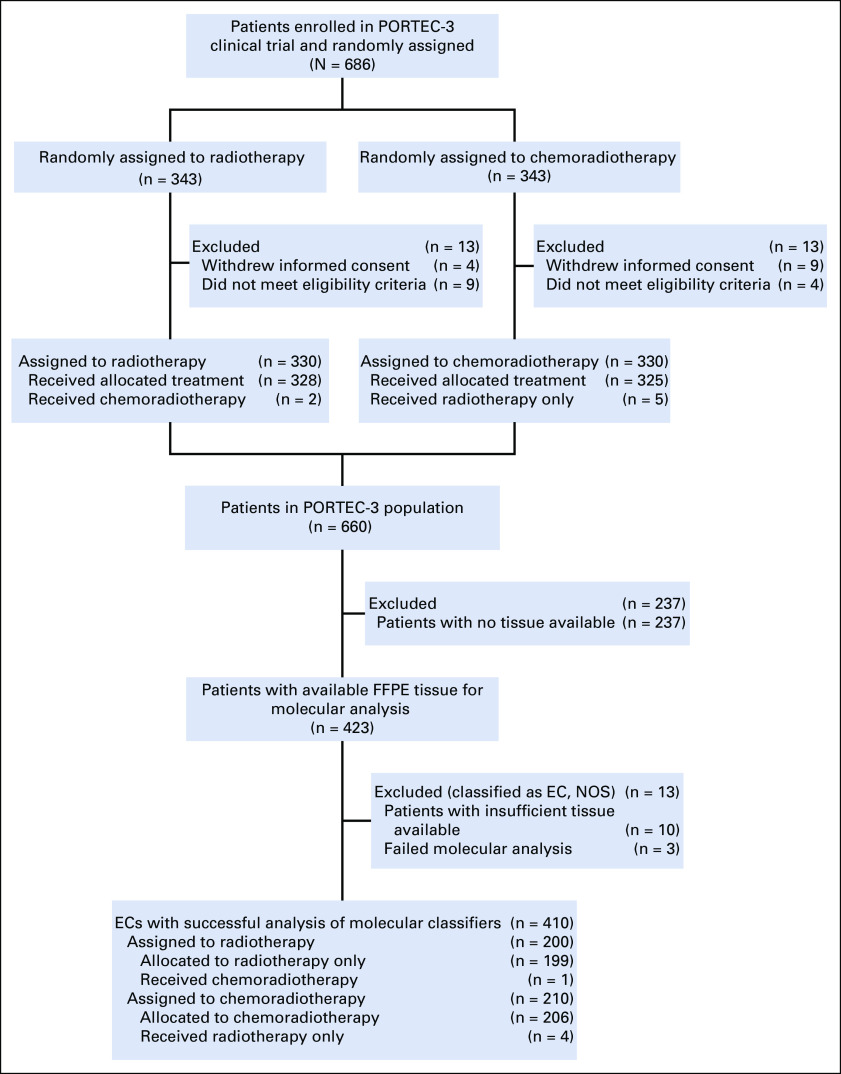
Flowchart of sample analysis. EC, endometrial cancer; FFPE, formalin-fixed, paraffin-embedded; NOS, not otherwise specified; PORTEC-3, Adjuvant Chemoradiotherapy Versus Radiotherapy Alone in Women With High-Risk Endometrial Cancer.

A total of 423 EC samples from PORTEC-3 participants were collected and available for molecular analysis. Molecular testing was successful for 410 tumors (97%; Fig 1). Patient and tumor characteristics of these 410 ECs were comparable to the trial population not included in this study (Data Supplement). Median follow-up was 6.1 years (range, 0.52-11.03 years).

The 410 ECs were classified in 1 of the 4 molecular subgroups (Data Supplement):

1. 93 (22.7%) were p53abn
2. 51 (12.4%) were *POLE*mut
3. 137 (33.4%) were MMRd
4. 129 (31.5%) were NSMP ECs.

Thirty multiple-classifier ECs were identified and allocated within 1 of the molecular subgroups (Data Supplement): 7 (1.7%) were *POLE*mut-p53abn, 9 (2.2) were *POLE*mut-MMRd, 11 (2.7%) were MMRd-p53abn, and 3 (0.7%) were *POLE*mut-MMRd-p53abn ECs. There were significant differences in age, histology, and stage among the molecular subgroups (Table 1). Of note, nonendometrioid ECs were found within all 4 molecular subgroups, as well as stage III cancers. The presence of LVSI, type of surgical procedure, and treatment (CTRT *v* RT) were well balanced among the molecular subgroups.

**TABLE 1.**
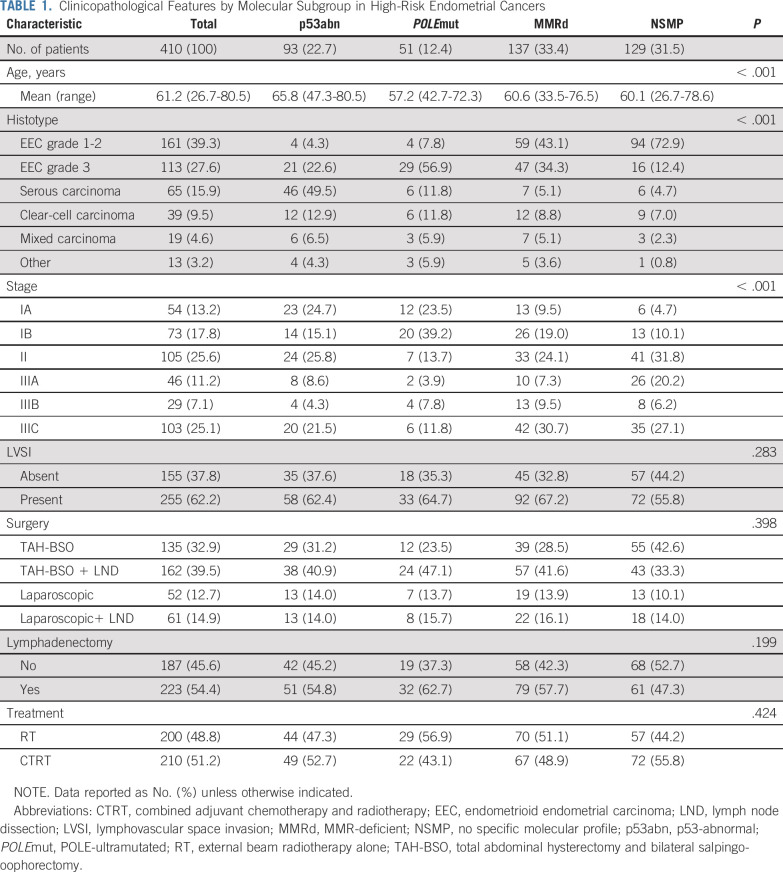
Clinicopathological Features by Molecular Subgroup in High-Risk Endometrial Cancers

### Clinical Outcome

**FIG 2.**
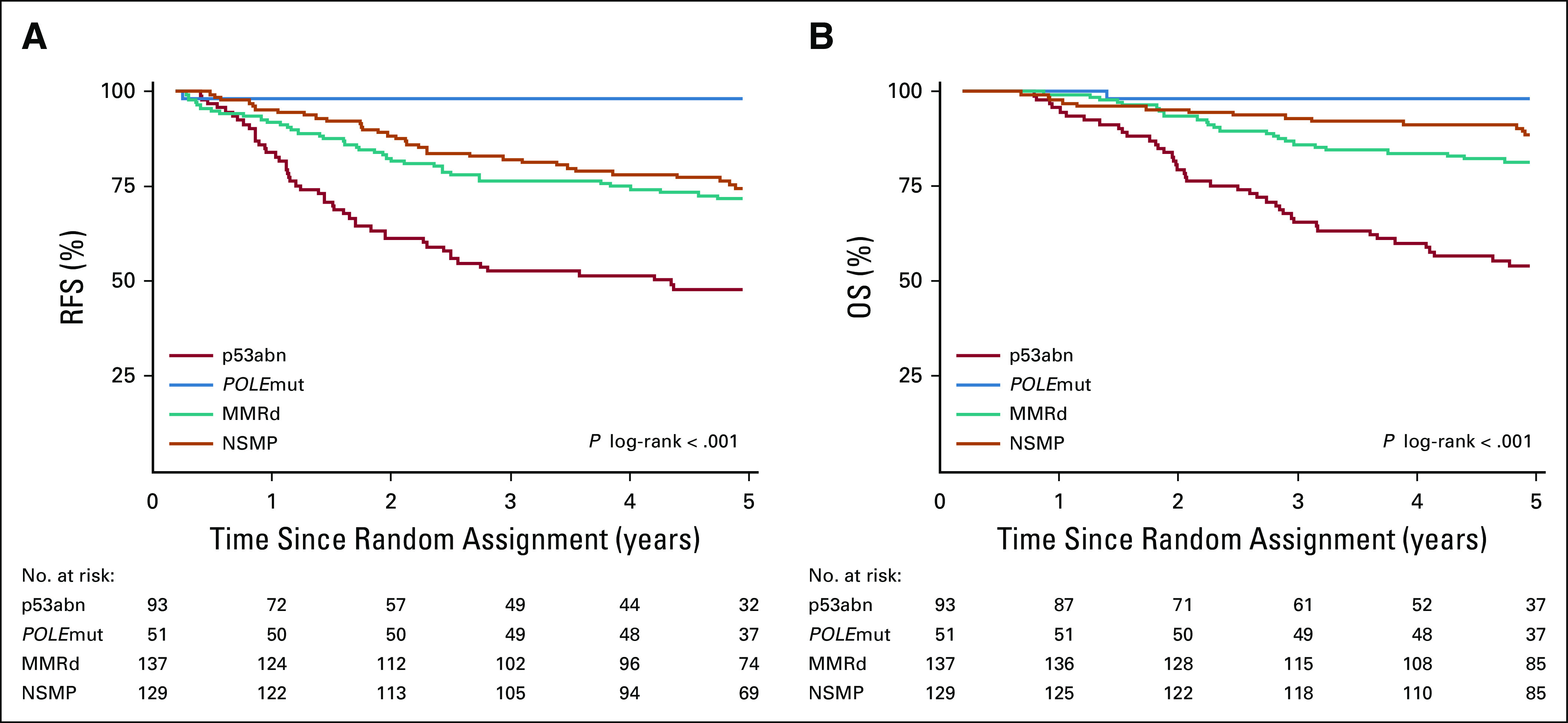
Kaplan-Meier survival curves for 5-year (A) recurrence-free survival (RFS) for patients with p53abn endometrial cancer (EC; 48.0%), *POLE*mut EC (98.0%), MMRd (71.7%), or NSMP EC (74.4%), and (B) overall survival (OS) in patients with p53abn EC (54.0%), *POLE*mut EC (98.0%), MMRd (81.3%), or NSMP EC (88.5%). MMRd, MMR-deficient; NSMP, no specific molecular profile; p53abn, p53-abnormal; POLEmut, POLE-ultramutated.

Patients with p53abn EC (n = 93) had the poorest prognosis, with 5-year RFS and OS of 48.0% and 54.0%, respectively. There were no statistically significant differences in clinical outcome between patients with p53abn serous type EC, and p53abn EC with other histologies (5-year RFS: 46.6% for serous p53abn *v* 48.9% for p53abn nonserous EC, *P* = .930; 5-year OS: 57.7% *v* 50.7%, *P* = .704). Five-year RFS and OS for women with *POLE*mut ECs (n = 51) were both 98.0%. Only 1 patient with a *POLE*mut EC had a recurrence and ultimately died of her cancer. Patients with MMRd (n = 137) and NSMP (n = 129) ECs had an intermediate outcome (5-year RFS: 71.7% and 74.4%, respectively; 5-year OS, 81.3% and 88.5%, respec-tively; Fig 2). All 30 patients with a multiple-classifier EC were alive without recurrence of their cancer at the time of data analysis (median follow-up, 5.3 years).

The prognostic value of the molecular EC classification was evaluated in univariable and multivariable analysis (Table 2). The p53abn subgroup was the strongest adverse prognostic factor, with a hazard ratio (HR) for RFS of 2.52 (95% CI, 1.62 to 3.91; *P* < .001) and HR for OS of 2.30 (95% CI, 1.42 to 3.73; *P* = .001), whereas *POLE*mut was the strongest favorable prognostic factor both for RFS (HR, 0.08; 95% CI, 0.01 to 0.58; *P* = .012) and OS (HR, 0.12; 95% CI, 0.02 to 0.87; *P* = .036; Table 2).

**TABLE 2.**
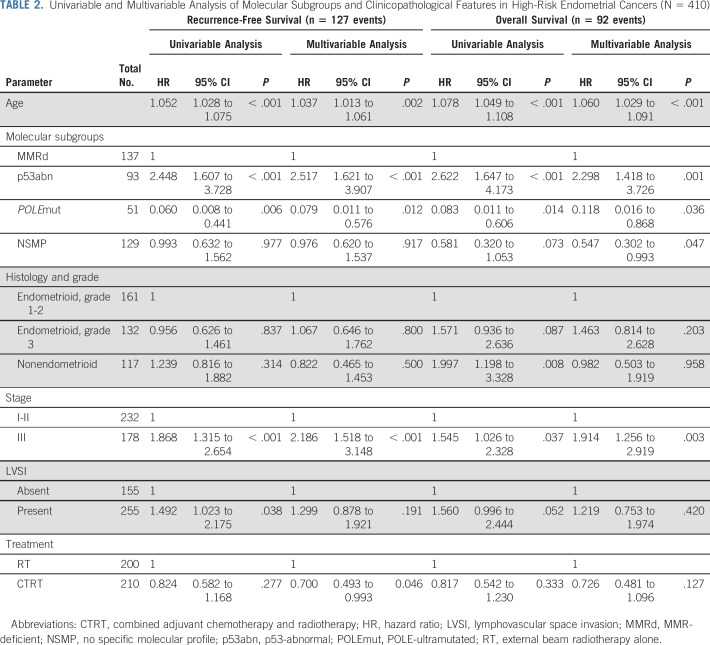
Univariable and Multivariable Analysis of Molecular Subgroups and Clinicopathological Features in High-Risk Endometrial Cancers (N = 410)

**FIG 3.**
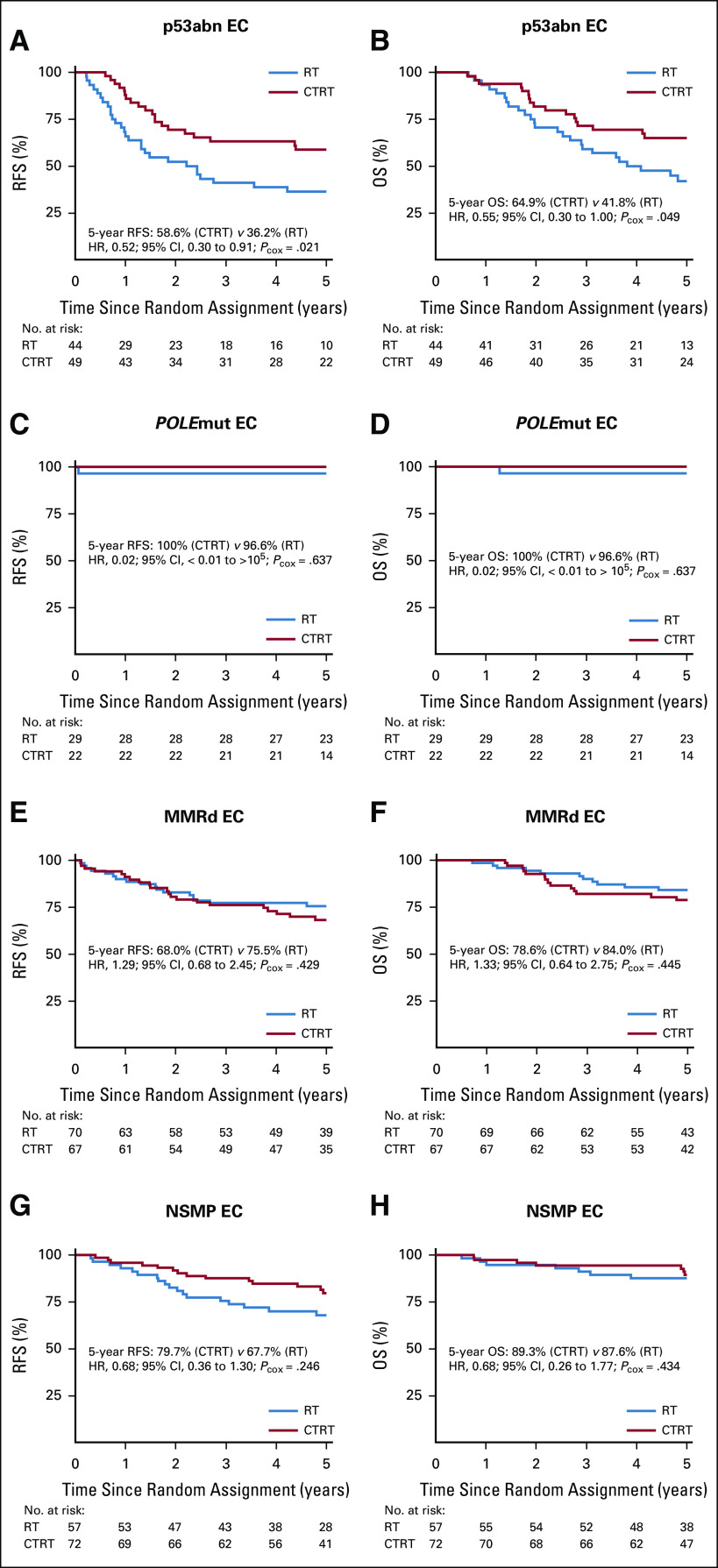
Kaplan-Meier survival curves for (A) recurrence-free survival (RFS) and (B) overall survival (OS) among patients with p53abn endometrial cancer (EC); (C) RFS and (D) OS among patients with *POLE*mut EC; (E) RFS and (F) OS among patients with MMRd EC; and (G) RFS and (H) OS among patients with NSMP EC. CTRT, combined adjuvant chemotherapy and radiotherapy; HR, hazard ratio; MMRd, MMR-deficient; NSMP, no specific molecular profile; p53abn, p53-abnormal; *P*_cox_, *P* value by Cox regression analysis; *POLE*mut, *POLE*-ultramutated tumor; RT, external beam radiotherapy alone.

We analyzed the differences in adjuvant treatment effect (CTRT *v* RT) among the molecular subgroups (Fig 3). Patients with a p53abn EC had statistically significant benefit from combined adjuvant CTRT, with an absolute difference of 22.4% for RFS (5-year RFS, 58.6% with CTRT *v* 36.2% with RT; HR, 0.52, 95% CI, 0.30 to 0.91, *P* = .021) and of 23.1% for OS (5-year OS, 64.9% *v* 41.8% for CTRT *v* RT; HR, 0.55, 95% CI, 0.30 to 1.00, *P* = .049; Fig 3). Test for interaction between molecular subgroups and treatment arm did not reach significance (RFS: *P =* .072; OS: *P* = .113). In exploratory subanalyses, the benefit from CTRT remained significant for RFS in early-stage p53abn EC but lost significance in stage III disease; however, numbers were small and testing for interaction between stage and adjuvant treatment in p53abn EC was not significant (Data Supplement).

Only 1 patient with a *POLE*mut EC (treated with RT alone) had disease recurrence, resulting in a 5-year RFS and OS of 100% with CTRT versus 96.6% with RT (HR, 0.02; 95% CI, < 0.01 to > 10^5^; *P* = .637; Fig 3).

Women with MMRd EC had a 5-year RFS of 68.0% with CTRT versus 75.5% with RT (HR. 1.29; 95% CI, 0.68 to 2.45; *P =* .429) and a 5-year OS of 78.6% with CTRT versus 84.0% with RT (HR, 1.33; 95% CI, 0.64 to 2.75; *P =* .445; Fig 3). Although patients with NSMP EC seemed to benefit from CTRT (5-year RFS: 79.7% with CTRT *v* 67.7% with RT; 5-year OS: 89.3% *v* 87.6%), this did not reach statistical significance (RFS: HR, 0.68, 95% CI, 0.36 to 1.30, *P =* .246; OS: HR, 0.68, 95% CI, 0.26 to 1.77, *P* = .434; Fig 3). Explorative subanalysis by stage within the patients with MMRd and NSMP EC did not reveal a benefit from adjuvant chemotherapy in advanced stages either (Data Supplement).

## DISCUSSION

To our knowledge, this study is the first to show the strong prognostic value of the molecular EC classification within a large cohort of patients with high-risk EC. Because of the randomized design of the PORTEC-3 trial, it is also the first, to our knowledge, to explore the potential predictive capacity of the molecular EC classification for benefit from chemotherapy. Patients with p53abn EC had a poor prognosis, in contrast to the excellent survival outcomes of patients with *POLE*mut EC, even among high-grade and advanced-stage cancers. Patients with MMRd or NSMP EC had an intermediate clinical outcome. Furthermore, patients with p53abn EC had a highly significant benefit from CTRT with an absolute benefit of 22.4% and 23.1% for 5-year RFS and OS, respectively, whereas patients with *POLE*mut EC had an excellent survival in both treatment arms. No benefit was observed from CTRT versus RT alone in patients with MMRd EC. Patients with NSMP EC had a trend toward benefit from CTRT, similar to the overall trial outcomes,^8^ but additional studies will be needed to elucidate the role of chemotherapy in this subgroup.

Indications for adjuvant treatment are currently based on well-established clinicopathological risk factors such as Federation Internationale de Gynecolgie et d'Obstetrique stage, grade, histologic type, LVSI, and age. Challenges with the current system include the lack of interobserver agreement in the evaluation of pathologic features, especially on histologic type in high-grade EC, where discrepancies reach 36% of cases examined.^9^ In contrast, the molecular EC classification is a highly reproducible system with strong prognostic value. The assessment of MMR proteins and p53 immunohistochemistry are highly concordant with MSI and *TP53* mutational status, respectively,^16,17^ and the interobserver agreement is > 95%.^17,18^ Authors of a recent study proposed pragmatic guidelines for the interpretation of nonhotspot *POLE*-EDM in EC,^13^ ensuring uniform interpretation. Furthermore, multiple-classifier EC can now be assigned to the appropriate molecular subgroup.^13,15^ The strong prognostic value of the molecular subgroups has been shown previously in high-intermediate risk EC,^3^ leading to additional studies to determine their role in adjuvant treatment. The ongoing, randomized PORTEC-4a trial compares standard adjuvant brachytherapy in women with intermediate-risk EC with individualized adjuvant treatment on the basis of the patients’ integrated molecular profile.^19^ Our results have shown strong prognostic value of the molecular classification even in high-risk and advanced-stage EC and regardless of histologic subtype, indicating that this classification should be incorporated in standard clinical diagnostics, treatment decisions, and future studies.

This study showed that patients with p53abn EC have a highly significant benefit from CTRT. These results are consistent with the PORTEC-3 clinical trial analysis, in which patients with serous cancers had a greater absolute benefit from CTRT.^8^ Indeed, 71% of serous cancers in the current study were classified as p53abn EC. However, the serous cancers that were molecularly classified as *POLE*mut (n = 6) and MMRd EC (n = 7) had no recurrences and patients were still alive at time of analysis. Only 2 of the 6 patients with a serous NSMP EC had a recurrence and ultimately died of their cancer. Additionally, 47 of 93 p53abn ECs (51%) had nonserous histology (23% EEC grade 3; 13% clear-cell cancers; 7% mixed; 4% grade 1 EEC; and 4% other histologies), and there was no difference in clinical outcome between this group and serous p53abn ECs. These results show that the molecular EC classification identifies a broader group of patients with a higher specificity who might benefit from CTRT compared with traditional histotyping.

To further improve the survival of patients with p53abn EC, research may be directed at the addition of targeted adjuvant treatment to CTRT. Recent studies have shown homologous recombination deficiency in p53abn EC,^20,21^ suggesting that these patients could benefit from the addition of poly(ADP-ribose) polymerase inhibitors to CTRT. Alternatively, for patients with p53abn EC and *HER2/neu* amplification (reported in 20%-25% of serous cancers^22^), trastuzumab (and possibly pertuzumab) in combination with chemotherapy is promising, as indicated by results of a recent phase II clinical trial.^23^

This analysis confirms the excellent survival of patients with *POLE*mut EC even in those with advanced-stage and nonendometrioid histologies, with no differences between adjuvant treatment received. This favorable clinical outcome is thought to be the result of the patients’ enhanced T-cell response due to their high mutational burden.^24^ Previous studies have described small groups of patients with *POLE*mut EC who received no adjuvant treatment and had no recurrences.^25,26^ Additionally, no increased sensitivity of *POLE*-mutant, mouse-derived embryonic stem cells to radiotherapy was found, nor to a selection of chemotherapeutics, including cisplatin.^25^ Together with our results, these data support the hypothesis that the excellent prognosis of patients with *POLE*mut EC is independent of adjuvant treatment. By implementing *POLE* testing in routine diagnostics, overtreatment of a substantial group of patients would be avoided, with clear impact on the patients’ quality of life.^27^

The lack of benefit observed from the addition of chemotherapy to RT in patients with MMRd EC suggests a favorable outcome with EBRT alone in stage I-II disease, as also found in the GOG-249 trial.^28^ Furthermore, a recent study reported benefit of early-stage MMRd EC from RT compared with no adjuvant treatment.^29^ Adjuvant treatment regimens other than chemotherapy should be explored in higher-stage MMRd ECs. Recently, immune checkpoint inhibition has been shown effective against MMRd solid cancers, which has led to US Food and Drug Administration approval of pembrolizumab for MSI cancers. Phase II clinical trials have shown high response rates to immune checkpoint inhibitors in advanced stage EC,^30^ which might be increased by the combination of these therapeutic agents with RT.^31^

The analysis of NSMP ECs showed only a trend toward benefit from adjuvant CTRT. Still, the HRs of CTRT versus RT for NSMP cancers were similar to those observed in the PORTEC-3 trial.^8^ The lack of significant effect of CTRT may be due to the relatively small numbers and molecular heterogeneity that characterizes NSMP EC.^1^ Additional studies directed at refinement of NSMP EC, such as characterization of *CTNNB1* exon-3 mutations, the potential fifth molecular subgroup,^3,32^ may elucidate which patients with NSMP EC might benefit from intensified adjuvant treatment.

PORTEC-3 analysis showed a greater benefit of added CT for patients with stage III EC and those with serous cancers.^8^ The current study provides insight into the biologic origins of this observation, identifying differences between the molecular subgroups. The high benefit of added CT observed across patients with p53abn EC in all stages (RFS: HR, 0.52; *P* = .022) corresponds partly to the benefit seen in serous cancers. The small numbers available per stage suggest that larger cohorts may identify significant differences in stage III p53abn EC (a nonsignificant benefit from CTRT was seen in this group of patients; RFS: HR, 0.58, *P* = .172), as well as in stage III NSMP cancers (RFS: HR, 0.68, *P* = .246). We observed no significant benefit from CTRT for patients with MMRd EC, even after analysis by stage, indicating that this molecular subgroup may not be contributing to the stage III results observed in PORTEC-3.^8^ Finally, although data were limited, patients with stage III *POLE*mut EC appeared to have a good clinical outcome in both treatment arms: only 1 of 12 patients randomly assigned to the RT arm had a recurrence and died of EC. Prospective observational studies will help define adequate de-escalation of adjuvant treatment of *POLE*mut EC, especially for stage III cancers.

Our study has limitations. Although it was a predefined translational research analysis within the context of the PORTEC-3 clinical trial, it was not originally powered for analysis by molecular subgroup. Additionally, we were able to obtain molecular results of 62% of the trial population. Nonetheless, the characteristics of the included cases were comparable to those of the excluded trial population.

In conclusion, our study shows the strong prognostic information the EC molecular classification carries, as well as its great potential to guide adjuvant treatment. It is essential to implement the molecular EC classification in clinical diagnostics and decision-making. Patients with p53abn EC may be considered for adjuvant treatment including chemotherapy, whereas adjuvant treatment de-escalation should be considered for those with *POLE*mut EC; additional studies are needed especially for MMRd and NSMP EC. Future clinical trials should include molecular subgroups in their design and study specific targeted adjuvant treatments.
